# Philosophical Physiology

**Published:** 1831

**Authors:** 


### XXXVI.

PHILOSOPHICAL PHYSIOLOGY. By M. BLAUD, M. D. 3 vols. 8vo. 1830.

[Review.]

Dr. BLAUD, who is a clever physician, and chief medical officer of the hospital Beaucaire, has published an elementary system of philosophical physiology, which will probably excite more interest in France than in England, on two accounts—its spirituality, and its opposition to Broussaism. M. Blaud attempts the study of life as connected with an immaterial soul—of organised structures as connected with intellect. He blames the physiologists for not availing themselves of the light of metaphysics—and he equally censures the metaphysicians for overlooking the assistance of physiology. He himself proposes to unite the two studies. One of his volumes is devoted to an investigation of the intellectual faculties, the material organs, and the condition of the existence of man. We can do little more than point out the results at which he has arrived.

The faculties of man are superemi-nently intellectual and moral. He thinks, wills, speaks, acts, believes, hopes, foresees, invents:—he is born for society. The author then examines the fabric of man, and finds it composed of material organs, "whose various functions combined constitute life." The nature of life escapes our investigation; but yet he judges it to be material, because it is perishable, and cannot apparently exist except in some mysterious connexion with matter. But if the principle of life be material, it is not so, according to our author, with the intellectual faculties of the soul. The soul is always denominated by him MAN par excellence. He argues that it is impossible for the intellectual faculties of man to belong to the encephalic apparatus, or brain. He denies that the brain can think, compare, judge, foresee, and make all those combinations of complicated operations which we see. The brain, he remarks, is a material organ composed of various parts, and too heterogeneous to perceive with *unity*. It is a complicated organ, and every idea, every perception is a unity — every image, every sensation is simple. This is the first cause of incompatibility—the first argument of the author.

II. Perception, says he, is active, voluntary, free. *Attention* is a free act of great importance. Abstraction is a work of choice, by which the soul or spirit concentrates its powers and discrimination on a body, on the quality or attribute of a body—on an idea, to the exclusion of all other subjects. In such an operation there is perfect freedom of choice; but the brain is a passive organ, which receives impressions through the senses without the power of quickening, retarding, or suspending the said impressions. This is another incompatibility, according to our author.

III. M. B. cannot imagine that the brain can perceive or think by means of motion, imprint, secretion, or any kind of irritation. None of these movements or imprints have ever been observed, and therefore they are gratuitous assumptions without any proofs.

IV. But supposing that the brain received and *discerned* the impressions which are transmitted by the senses—real impressions, commemorative images of material objects, how is it to receive from *words* a true image of real objects, of which these words express the memory, the nature, or the attributes? Can a mass of organized matter transform a simple vibration of the tympanum or chordæ vocales, a motion of the tongue or lips into real objects, or into abstract thoughts expressed by articulate sounds?

V. "I am conscious," says M. Blaud, "that I perceive. I perceive, therefore, my own personal impressions. But can such an action appertain to the encephalon? In such case, the encephalon must re-act on itself. Matter is incapable of *such* re-action. Organs act and re-act on each other, in consequence of impressions received from one another; but this kind of re-action is free, spontaneous, arbitrary."

VI. Besides our perceptions often appear independent of actual impressions. Our presentiments, fears, hopes, are often altogether unconnected with any sensations at the moment.

For these and many other reasons M. Blaud concludes that it is not the brain which conceives ideas or appreciates sensations—which compares, judges, remembers, or imagines. "None of the faculties of man reside in the encephalic mass. Man (by which M. B. designates the soul) is a being essentially immaterial; though it cannot exercise its faculties but by the medium of this encephalic mass. In this respect it is, in a certain degree, dependent on or connected with the brain."

These sentiments have long been maintained in this country; but they have been very sparse in France, since the revolution, 1789. Some of the French critics insinuate that this book was composed for a certain religious society in France, called "La Société des bons Livres," from which society it gained a prize; and that it is now put forth for general circulation, without any allusion to the original purpose for which it was designed, and without any mention of the honours which it gained. These are rather suspicious circumstances, and may be connected with the recent downfal of regal and papal fanaticism in the "Grande Nation." The same critic concludes thus:—"the work which we announce has for its author a studious man—a physician zealous in his profession—an intelligent writer, whose misfortune it is to have passed too much of his time far from capitals, *the only places where alone good books can be constructed.*" These reflections will sound rather strange in English ears, when we remember the works of a Currie, a Darwin, a Parry, a Hey, and a hundred other authors. Were the metropolitan writers on medicine, indeed, to be ranged in comparison with the provincial, of England, the former would cut a very sorry figure. Yet the observation of the French critic is, no doubt, correct, as far as France is concerned. Paris is France—and France is only an inconsiderable appendage.

---

